# The Role of the Heat-Shock Proteins in Esophagogastric Cancer

**DOI:** 10.3390/cells11172664

**Published:** 2022-08-27

**Authors:** Francisco Tustumi, Gabriel Andrade Agareno, Ricardo Purchio Galletti, Rafael Benjamim Rosa da Silva, Julia Grams Quintas, Lucas de Abreu Sesconetto, Daniel José Szor, Nelson Wolosker

**Affiliations:** 1 Department of Gastroenterology, Universidade de São Paulo, Av. Dr. Enéas Carvalho de Aguiar, 255, São Paulo 05403-000, SP, Brazil; 2 Department of Surgery, Hospital Israelita Albert Einstein, Av. Albert Einstein, 627, São Paulo 05652-900, SP, Brazil

**Keywords:** heat-shock proteins, esophageal neoplasm cancers, stomach neoplasm

## Abstract

Heat-shock proteins (HSPs) are a family of proteins that have received considerable attention over the last several years. They have been classified into six prominent families: high-molecular-mass HSP, 90, 70, 60, 40, and small heat shock proteins. HSPs participate in protein folding, stability, and maturation of several proteins during stress, such as in heat, oxidative stress, fever, and inflammation. Due to the immunogenic host’s role in the combat against cancer cells and the role of the inflammation in the cancer control or progression, abnormal expression of these proteins has been associated with many types of cancer, including esophagogastric cancer. This study aims to review all the evidence concerning the role of HSPs in the pathogenesis and prognosis of esophagogastric cancer and their potential role in future treatment options. This narrative review gathers scientific evidence concerning HSPs in relation to esophagus and gastric cancer. All esophagogastric cancer subtypes are included. The role of HSPs in carcinogenesis, prognostication, and therapy for esophagogastric cancer are discussed. The main topics covered are premalignant conditions for gastric cancer atrophic gastritis, Barrett esophagus, and some viral infections such as human papillomavirus (HPV) and Epstein–Barr virus (EBV). HSPs represent new perspectives on the development, prognostication, and treatment of esophagogastric cancer.

## 1. Introduction

In 1962, Professor Ferruccio Ritossa, an Italian geneticist investigating proteins produced with sudden heat in *Drosophila busckii*, described the heat shock proteins (HSPs) [1]. At that time, it was thought that the release of HSP would occur only with an abrupt change in temperature, which was coined heat shock response. Subsequently, HSPs were identified as a typical response to a long list of stresses, such as oxidative stress, nitrosative stress, pH change, fever, and inflammation [2].

Initially, Professor Ferruccio Ritossa’s findings were considered irrelevant [3]. However, the number of implications related to the heat response of HSPs has risen exponentially over the decades [3]. Initially described in *Drosophila busckii,* HSPs functions have been found throughout all kingdoms. The structural organization of these proteins shows conservative features across the species [4].

Heat-shock proteins are a group of proteins that reverse or inhibit denaturation or cellular proteins unfolding in response to any deviation or process affecting homeostasis, including high temperature, hypoxia or anoxia, heavy metals, drugs, or other chemical agents that may trigger protein unfolding [5]. HSPs facilitate protein folding and maintenance of other proteins’ natural structures and functions in stressful environments [5,6]. HSPs are also known as molecular chaperons because of their protective roles in cells, working as biochemical regulators to mediate cell growth, apoptosis, protein homeostasis, and cellular function [7].

Under heat shock conditions, heat shock factors (HSF) mediate a transcriptional response, dissociating of HSPs and binding to heat shock elements (HSEs) to activate specific genes, which lead to HSPs’ expression [8].

HSPs are classified according to their molecular weights (kDa) and function. The main HSP families are the small HSPs (≤34 kDa), HSP 40 (35 to 54 kDa), 60 (55 to 64 kDa), 70 (65 to 80 kDa), 90 (81 to 99 kDa), and high-molecular-mass HSPs (≥100 kDa, such as HSP 110 and glucose-regulated protein 170, GRP 170) [9]. Most of them can convert ATP into ADP [10].

Abnormal expression of HSPs has been found in numerous medical conditions, including autoimmune disorders, cardiovascular problems, skin conditions, and organ transplants [11,12,13,14]. Additionally, HSPs may play significant roles in the molecular mechanisms leading to cancer development and progression [15]. The expression of HSPs has been associated with cancer cells’ survival and progression, cellular differentiation, apoptosis, cellular functions, carcinogenic pathways, and cancer invasiveness and dissemination [16].

Among the several different types of neoplasms, esophagogastric cancer is the second leading cause of cancer-related death worldwide [17]. Although survival has improved in the last years, the overall prognosis remains poor, with high recurrence rates and a lack of efficient systemic therapy [18]. The diagnosis of esophageal and gastric cancer is usually made when the patient already has a locally advanced or systemic disease due to the low occurrence of signs and symptoms in the early phase [18]. Investigating and exploring novel biomarkers that may help understand esophagogastric carcinogenesis, stratify prognosis, and guide therapy is critical to improving outcomes in this disease. 

In this sense, the present study aims to review all the evidence covering the role of HSPs in the pathogenesis, prognosis, and their potential role in future treatment options for esophagogastric cancer. 

## 2. Methods

A systematic literature search was performed for medical evidence regarding HSPs related to esophagus and gastric cancer. All gastric and esophageal cancers were included. The following search terms were used: 

”Heat Shock Proteins”, ”HSP”, “Heat Shock”, “Esophagus”, “Esophageal”, “Esophagectomy”, “Oesophagus”, “Oesophageal”, “Oesphagectomy”, “Gastric”, “Stomach”, “Esophagogastric”, “Cardia”, “Cancer”, “Neoplasm”, “Tumor”, “Preneoplastic”, and synonyms. The main databases searched were PubMed, Embase, Lilacs/BVS, Cochrane Central, and Google Scholar. Any observational or experimental human study and animal models were included.

## 3. Literature Review

After the literature search, we identified several HSPs associated with esophagogastric cancer development, prognosis and that have role in future anti-cancer treatment modalities. 

### 3.1. Role of HSP in Carcinogenesis of Esophagogastric Cancer

Heat shock proteins may play numerous roles in regulating cancer development. The stressful changes within the tumor microenvironment, including reducing glucose, oxygen, and acidification, may instigate HSP expression [19]. However, the precise mechanisms have not yet been determined, although they likely involve molecular changes common to an extensive range of cancer types, causing the heat shock response activation [19,20].

Noguchi et al. [21] investigated the function of HSP 70 as a chaperone for abnormal p53 expression, which is very frequent during carcinogenesis of esophageal squamous cell carcinoma. However, the authors found no correlation between HSP 70 and p53. Likewise, Maehara et al. [22] found that the HSP 70 family expression and abnormal p53 staining are not correlated in gastric adenocarcinoma tissues. On the other hand, a Japanese study with 182 patients submitted to curative intent gastric resection for cancer investigated specifically the mortalin, a stress chaperone that belongs to the HSP 70 family [23]. This study described a robust correlation between mortalin and aberrant p53 [23]. In a canine gastric cancer investigation, HSP 27 presented a robust negative association with p53 indices [22]. In addition, in this study, HSP 27 expression had a higher mean p21 expression than those with low HSP 27 expression (47.4% vs. 25.7%).

Gastroesophageal reflux disease and Barrett’s esophagus are important risk factors for esophageal and cardia adenocarcinoma development [24]. Consequently, the role of HSPs in carcinogenesis of those neoplasms depends substantially on understanding their role in Barrett’s and esophageal reflux. A study of reflux esophagitis with an animal model [25] showed that HSP 27 mRNA expression is higher within the distal esophagus of rats with esophagitis than in controls. Conversely, the expression of HSP 70 is reduced after thermal injury to the esophageal epithelium [26]. Succeeding esophagitis recovery, HSP 70 increases [26].

**Figure 1:**
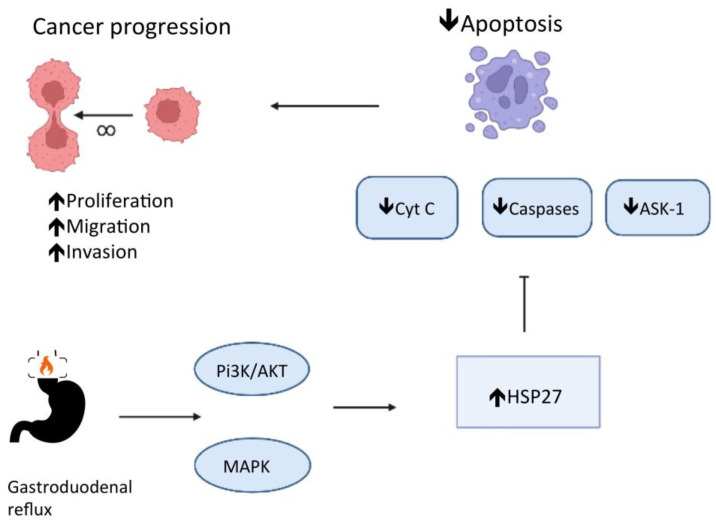
HSP 27 role in the deregulation of apoptosis and carcinogenesis in chronic esophagitis.

Phosphatidylinositol 3-kinase (PI3K) and p38 mitogen-activated protein kinase (MAPK) regulate the expression of Hspb1, the HSP 27 gene, in cultures of esophageal endothelial cells in response to esophageal acid exposure [27]. The low pH in the esophageal lumen promotes the phosphorylation of PI3K and MAPKs, which catalyze the phosphorylation of HSP 27 [27,28], resulting in an HSP 27 remodeling, turning large oligomers into small units [29]. HSP 27 also interacts with the protein kinase B (Akt) and blocks the cytochrome c (Cyt C) release in the cytoplasm. Both Akt and Cyt C have significant roles in cell apoptosis [29]. HSP 27 also blocks apoptosis by regulating the apoptosis signal-regulating kinase 1 (Ask1), a member of the MAPK family, and the Fas receptor (CD 95) function, a cell surface death receptor [29]. In addition, HSP 27 has been reported to reduce reactive oxygen species (ROS) accumulation. Consequently, HSP 27 protects cells from damage and blocks apoptosis [30] through several pathways and is a mediator of esophageal epithelial cell proliferation [28]. Deregulation in apoptosis is a well-known crucial step for carcinogenesis [29] (see Figure 1).

Although the HSP 27 has a significant role in esophagitis, Zhang et al. [31], evaluating patients with esophagitis with and without Barrett’s esophagus, showed that the expression of HSP 27 seems to remain unaltered [31]. However, patients with Barrett’s esophagus had significantly lower expression of HSP 70 and HSP 90α than patients with esophagitis without Barrett’s esophagus [31]. In Zhang et al.’s study [31], the telomerase reverse transcriptase (TERT) expression was also reduced in Barrett’s esophagus. The HSP 105 and the Caspase-3 expressions are increased when comparing Barrett epithelium to esophagitis without Barrett’s [31]. The findings of Zhang et al.’s study [31] may suggest some insights. 

**Figure 2:**
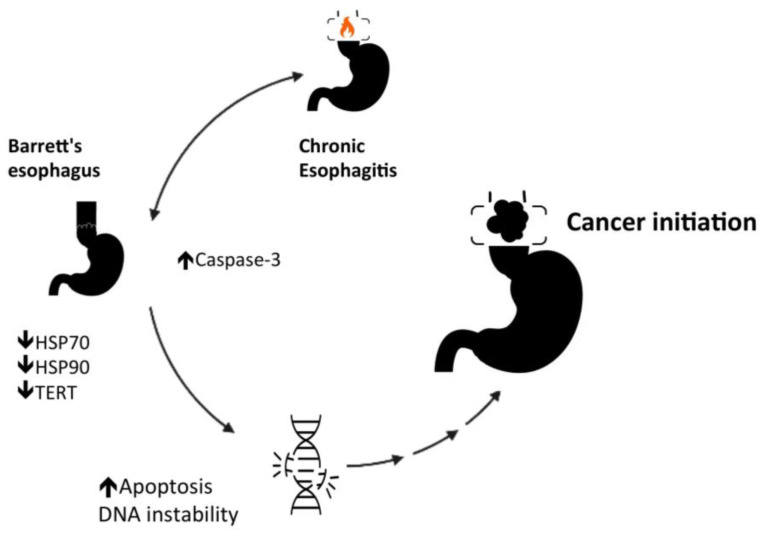
Cancer initiation in Barrett’s esophagus.

Telomerase activity has a close relationship with telomere length and cell survival [32]. In Barrett’s esophagus, the TERT expression reduction associated with the increased expression of Caspase-3, which has a central role in apoptosis, suggests that Barrett’s epithelium may be a microenvironment prone to DNA instability (see Figure 2). HSP 70 and 90α could counteract oxidative stress, but their low expression in Barrett’s esophagus may be prone to chronification of the preneoplasic condition. However, the increase in HSP 105 expression can represent a cytoprotective mechanism in Barrett’s epithelium. Previous studies have shown that the upregulation of HSPs counterbalances inflammation and oxidative stress, preventing lipid peroxidation and perturbation of the mucosal-barrier integrity [30,33]. The proliferation and apoptosis could be heterogeneous along Barrett’s epithelium. Therefore, the evolution of cancer could rely on a network of various carcinogenic pathways. 

Chronic atrophic gastritis is a premalignant condition for gastric cancer [34]. The gastric mucosa’s long-term inflammatory condition promotes metaplasia, dysplasia, and cancer development [34]. Animal and human studies report a significant correlation between chronic atrophic gastritis and abnormal HSP 27, 70, and 90 [35,36,37]. The HSP 70 is upregulated with gastric mucosa inflammation [35]. After applying an HSP 70 inhibitor, such as quercetin, the antral inflammation accentuates, suggesting HSP 70 may have a cellular protective role in chronic atrophic gastritis [35]. In patients with chronic atrophic gastritis, the progressive increase in the expression of HSP 70 and 90 indicates the aggravation of the inflammatory condition and may help predict the development of intraepithelial gastric adenocarcinoma [36,37]. Gastric adenocarcinoma tissues express a lower HSP 70 expression than chronic atrophic gastritis tissues, suggesting that the HSP 70 cytoprotective role may have weakened during cancer development [36]. Nagata et al. [37] showed that patients with atrophic gastritis with intraepithelial neoplasia have significantly lower HSP 27 expression than patients without intraepithelial neoplasia. Moreover, the HSP 27 expression is higher for tumors with a poor grade of differentiation [29,37]. Probably, the heat shock proteins have two distinct stages in gastric carcinogenesis (see Figure 1 and Figure 2). HSP 27 regulates both gastric epithelium apoptosis and inflammation. At the outset, the loss of cytoprotective effect (HSP 27 downregulated) promotes cancer initiation, and subsequently, the loss of apoptotic effect (HSP 27 upregulated) promotes cancer progression [29,37]. In the last stage, HSP 27 facilitates recovery or prevents the destruction of proteins, promoting cancer cells’ survival [29,37].

Some viral infections are involved in the development of some neoplasms. The human papillomavirus (HPV) may promote a distinct microenvironment in esophageal squamous cell carcinoma [38]. HPV infection seems related to HSP 90 and 16.2 overexpression [39,40], enabling a microenvironment prone to DNA instability. 

Epstein–Barr virus (EBV) is found in 8.77% (95% CI 7.73 to 9.92) of people with gastric adenocarcinoma [41], and consequently, this infection has been attributed to a part of the carcinogenic process in some patients [42]. Epstein–Barr virus promotes the HSP 27 phosphorylation via the PI3K/AKT pathway [42]. Furthermore, the HSP 27 in EBV-positive cells is decreased after using PI3K inhibitors, such as wortmannin or LY294002 [42]. This data may provide future research lines in gastric cancer prevention for patients in high-risk groups, and HSP 27 may be a biomarker for tailored therapy.

### 3.2. Role of HSPs in Prognostication of Esophagogastric Cancer

Understanding and stratifying the cancer prognosis assists with medical decisions and sharing with the patients and their families. Proper prognostication avoids unnecessary treatments that might produce more suffering than benefits. The abnormal heat shock protein expression could influence cell proliferation, differentiation, invasion, metastasis, and anti-apoptotic activity and, consequently, could be associated with esophagogastric cancer prognosis [19,43].

In esophageal squamous cell carcinoma, HSP 27, 60, 70, and 90 seem unrelated to the risk for systemic metastasis (M stage) [21,44,45]. However, the HSP expression correlation with T and N stages is quite heterogeneous among studies. Some studies show a positive relation with lymph node dissemination [21,46], whereas others found no significant association [44]. Some studies found a positive association with tumor depth [46], whereas others found no association [44,45]. Future meta-analyses are required to determine the pooled risk ratio.

Some HSPs may help predict overall survival in esophageal squamous cell carcinoma. HSP 27 overexpression imposes a poorer long-term survival [46,47,48]. Nonetheless, HSP 16.2 and 70 are not considered independent predictors of overall survival [21,47,49].

In esophageal adenocarcinoma, the pretreatment tumor stage does not correlate with HSP 27, 70, and 90 expressions [50]. However, Söderström et al. [51] found that HSP 27 and HSP 70 overexpression could be a decisive negative predictive factor for long-term survival. Patients with high HSP 27 have a mean overall survival of 23 months, and patients with negative HSP 27 or low expression have 49 months mean overall survival [51]. Patients with HSP 70 high expression have significantly lower overall survival than patients with negative or low expression (17 vs. 40 months) [51].

For gastric adenocarcinoma, mortalin, a stress chaperone belonging to the HSP 70 family, has been described as an independent prognostic factor [23]. Mortalin-positive gastric tumors have deeper invasion and a higher risk for lymph nodal and liver metastasis than mortalin-negative tumors [23]. Additionally, mortalin is significantly related to long-term survival for gastric cancer [23]. Mortalin binds to p53 and prevents expected apoptosis and tumor suppression [23]. Therefore, future molecule-targeting treatment against mortalin may provide new therapeutic tools for gastric cancer [23].

Kapranos et al. [52] described the variation in HSP 27 expression among different gastric epithelial tissues. HSP 27 overexpression was more frequent in the dysplastic gastric epithelium, and the expression increased with epithelial dysplasia severity. In addition, HSP 27 was related to lymphatic dissemination and shorter overall survival in univariate analysis but not in the multivariate analysis [52].

Zhai et al. [53] evaluated the prognostic value of HSP 70/HSP 90-organizing protein (HOP), an auxiliary protein that regulates HSP 70 and 90 folding in gastric cancer. High HOP protein expression in gastric tissues was related to advanced Borrmann classification, grade of cellular differentiation, tumor invasiveness, lymph nodal dissemination, and metastasis. Survival analysis demonstrated that patients with high HOP expression had shorter overall survival than those with low expression [53]. Table 1 summarizes the main heat shock proteins with their corresponding impact on survival in esophagogastric cancer.

### 3.3. Role of HSP in New Treatments for Esophagogastric Cancer

Investigating HSP-based drugs for cancer immunotherapy is another subject of increasing interest. The cancer cells’ escape from the immune system is a crucial step during cancer development [54]. Immunotherapy is an anti-cancer strategy that promotes immunogenic activity in the neoplasm cells and helps the immune system fight against cancer [54,55]. Cancer immunotherapy relies on triggering the immune system to promote a self-sustained effect against cancer cells without stimulating an immune response against normal host cells. Novel immunotherapy strategies have gained recognition for treatment of numerous cancer types, such as lymphoma, melanoma, colorectal adenocarcinoma, pancreatic cancer, glioblastoma, renal cell carcinoma, and gastric adenocarcinoma [56].

The main mechanisms for cancer immunotherapy comprise immune checkpoint inhibitors, T-cell transfer therapy, monoclonal antibodies, immune system modulators, and treatment vaccines [55,56]. 

Novel discoveries suggest that HSP-based vaccines can promote enhanced stimulation to tumor cells and more efficient antigen presentation to CD4+ and CD8+ T cells [57]. Certain heat shock protein domains present a significant immunogenic target for adaptive immunity, such as the ATPase domain of some members of the HSP 70 and 90 families [56]. Consequently, exogenous heat shock protein-related peptide immune complexes with high immunogenic effect could elicit a response against cancer cells and work as anti-cancer therapy.

Shimizu et al. [58] conducted a phase I clinical trial investigating HLA-A2- and HLA-A24-restricted HSP 105 peptide vaccines in patients with esophageal and colorectal cancer. The authors found that HSP 105-specific cytotoxic T-lymphocytes induction may improve progression-free survival and overall survival.

A non-randomized phase II clinical trial [59] investigated the effect of a vaccination based on a glycoprotein with a molecular weight of 96 kDa (gp96) as an adjuvant therapy for gastric adenocarcinoma. gp96 is a member of the HSP 90 family with ATPase activity [56]. In this clinical trial, the disease-free survival was higher in the group receiving vaccination plus chemotherapy than chemotherapy alone.

Her 2 testing has become one of the cornerstones in recent immunotherapy for gastric and gastroesophageal junction adenocarcinoma [60]. In addition, amplification of Her 2 is related to a more aggressive biological behavior [61]. Her 2 activity is modulated by molecular chaperones such as HSP 90 [50]. Deregulated HSP 90 expression may represent a possible resistance mechanism to Her 2 targeted drugs [62]. Berezowska et al. [63] showed a significant correlation between Her 2 and HSP 90 expressions in gastric cancer. Studies in mammary cells indicate that HSPs contribute to Her 2-induced carcinogenesis [64,65]. These findings may indicate a synergistic regulation between HSP and Her 2. Consequently, future trials targeting heat shock proteins and Her 2 may improve immunotherapy efficacy for esophagogastric adenocarcinoma treatment. 

The HSP 70 protein is also used to stimulate natural killer cells or by introducing HSP70 mRNA into cells (transfection) to elicit an immune response against tumors [56]. HSP70 mRNA-transfected dendritic cell therapy has been studied in phase I/II studies for hepatocellular carcinoma [66]. The HSP 70 TKD peptide and interleukin-2 have been studied to activate autologous natural killer cells [56]. The HSP 70-targeting activated natural killer cells approach has been studied as another immunotherapy strategy for glioblastoma multiforme and lung cancer [56]. No clinical trial using the HSP 70-targeting activated natural killer cells or transfection approaches for esophagogastric cancer has been published. 

The overexpression of some heat shock proteins within the cancer tissue suggests the potential for therapy based on these proteins. HSP inhibitors may act as potential drugs for cancer downstaging, and HSPs may also work as biomarkers for response prediction to neoadjuvant therapy. Various HSP inhibitors are being tested in preclinical studies and clinical trials for esophagogastric cancer. The inhibition of HSP proteins could theoretically block cancer development with minimal toxicity to normal tissues, which usually do not overexpress these proteins. This strategy is named target therapy and is one of the main goals of contemporary oncology [67].

Currently, the HSP 90 inhibitors are the most studied chaperone targets for anti-cancer therapy [68]. HSP 90 plays a central role in regulatory pathways such as cell signaling, apoptosis, and the cell cycle [69]. These abundant chaperones are highly conserved and participate in critical functional cellular processes [69]. HSP 90 interacts with proteins that participate in the carcinogenesis checkpoints, such as the signal-transduction enzymes, apoptotic proteins, transcriptional factors, and an extensive range of other cell cycle and oncogenic proteins [70]. HSP 90 protein contributes to the maturation and stabilization of the telomerase [70]. In addition, the HSP 90 protein in cancer tissues shows a significantly higher affinity for inhibitors [71]. These data indicate that HSP 90 inhibition has potential use as a targeted therapy for esophagogastric management. The HSP 90 inhibitor binds to HSP 90 and prevents adequate client protein folding. Consequently, the HSP 90 inhibitor leads to the degradation of the client protein via the proteasome pathway [71]. 

The main HSP 90 inhibitors are the geldanamycin analogs, resorcinol derivatives, and purine analogs [71]. In a preclinical study, Vesci et al. [72] investigated the antitumor activity of SST0116CL1, an HSP 90 inhibitor. The authors concluded that SST0116CL1 effectively inhibited cell growth in solid tumors, including gastric cancer. A phase-2 clinical test [73] that investigated ganetespib (STA-9090), another HSP 90 inhibitor, has not shown a significant therapeutic response in patients with esophagogastric cancer. However, the study included only advanced tumors refractory to the traditional therapy, and the small sample size (N = 26) limited the power analysis. The most frequent ganetespib adverse events were diarrhea, fatigue, elevated alkaline phosphatase, and elevated aspartate transaminase. Wang et al. [74] investigated the BIIB021, an HSP 90 inhibitor, and found that BIIB021 sensitized esophageal squamous cell carcinoma cells to radiation. 

Another contribution of the heat shock proteins to esophagogastric therapy is their potential role as biomarkers for predicting the response to neoadjuvant therapy. Zoltan et al. [49] evaluated the pretreatment expression HSP 16.2 in esophageal squamous cell carcinoma biopsies. The authors found that the expression levels of HSP 16.2 were significantly correlated with poor clinical and pathological responses. In another esophageal squamous cell carcinoma study [75], tumors with no complete pathological response to neoadjuvant therapy expressed twice the levels of HSP 90 and HSP 16.2 as tumors with a complete pathological response. Similarly, Bognár et al. [39] showed that HSP 16.2 and 90 overexpressing tumors are less likely to show clinical downstaging after neoadjuvant therapy. Langer et al. [76] evaluated the neoadjuvant therapy with platin and 5-fluorouracil for esophageal adenocarcinoma. The patients with response to neoadjuvant therapy had higher HSP 27 expression. However, HSP 60 showed a non-significant value for predicting pathological response to therapy [76].

The main potential therapies targeting each class of HSP and their molecular mechanism of action are summarized in Table 2. 

### 3.4. Perspectives for HSP in Esophagogastric Cancer

Therapy against the most diverse types of cancer has steadily evolved [77,78]. Studies on cellular and molecular mechanisms make treatment more effective and less harmful to health. HSPs are closely involved in cancer development, progression, and response to therapy; consequently, HSPs may be the next step in improving the quality of esophagogastric cancer management [15]. Knowledge of HSPs’ roles will allow better medical decision-making by customizing management according to the patient’s prognostic and molecular cancer characteristics. 

Personalized medicine and precision therapy are some of the paramount issues of modern oncology [79]. The HSP biomarker profile of cancer tissues may help indicate specific palliative, adjuvant or neoadjuvant chemotherapy or radiotherapy schemes. Future molecular pathology routine testing in esophageal and gastric neoplasms may help predict overall survival and facilitate grouping patients according to their probability of response to chemoradiotherapy. Patients matched with targeted therapy have more prolonged overall survival and lower treatment costs than non-matched patients [80,81,82].

Searching for a complete response after chemoradiotherapy for a watch-and-wait approach is one of the primary goals for esophageal cancer, mainly squamous cell carcinoma [83]. Knowing the expected response to chemoradiotherapy may allow some patients to benefit from avoiding an esophagectomy after chemoradiotherapy. Heat shock protein profiling will improve precision in esophageal cancer management and may help select patients with a higher likelihood of complete response to chemoradiotherapy.

Future therapies with HSP inhibitors and HSP cancer vaccines may improve survival and interrupt cancer initiation and progression. Currently, most HSP inhibitors studies are restricted to preclinical analysis or early clinical tests. HSP inhibitors and immunotherapy may change the way we treat advanced esophagogastric neoplasms. Several immunotherapy-based drug schemes are used to treat metastatic esophageal or gastric cancer [84,85]. However, most of these immunotherapy agents are PD-L1-based. Anti-cancer HSPs vaccines may be added to the current therapeutic arsenal for advanced esophageal and gastric cancer. Furthermore, the type of neoadjuvant therapy may also change if HSPs’ vaccines significantly affect cancer downstaging. Immunotherapy as preoperative therapy for esophageal cancer has already been studied with PD-L1 based-schemes [86]. Preclinical studies suggest neoadjuvant immunotherapy may even be superior to adjuvant treatment in eradicating micrometastases [87].

Heat shock proteins may participate in each carcinogenesis hallmark, such as initiation, promotion, and progression. Theoretically, understanding the HSP role in carcinogenesis may enable blocking of carcinogenesis checkpoints and contribute to cancer prevention in high-risk groups, comprised patients with hereditary syndromes or preneoplastic conditions, such as Barrett esophagus, atrophic gastritis, HPV or EBV viral infections [41,88,89,90].

**Figure 3:**
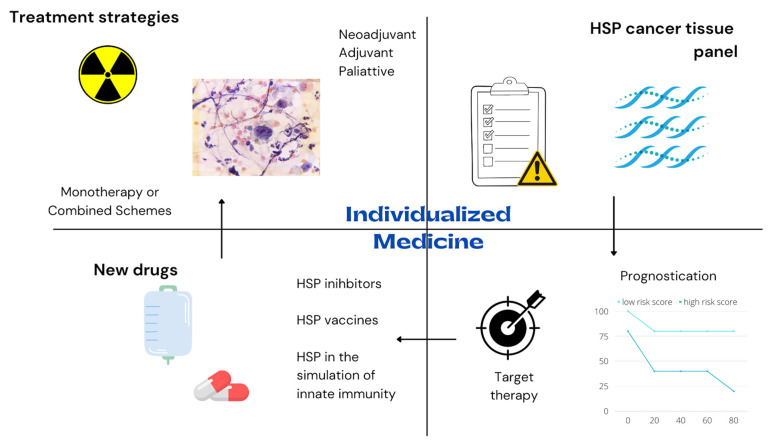
HSP roles for the future management of esophagogastric cancers.

Future studies on HSPs for esophagogastric cancer are imperative. The efficiency and security of HSP-based cancer vaccines and HSP inhibitors should be investigated in randomized phase III clinical trials to allow the introduction of these drugs into the market and to bulk the arsenal of esophagogastric cancer therapy as monotherapy or as novel combinatorial strategies. In addition, the role of HSP as molecular biomarkers should also be investigated in future studies to allow better oncologic patient risk stratifications. In the future, the HSP panel profile of the esophagogastric tumor should be used in risk calculators for predicting cancer progression and helping decision-making and customized management. See Figure 3. 

## 4. Conclusions

Heat shock proteins represent new perspectives on understanding esophageal and gastric cancer development and progression. The findings from heat shock protein studies will help improve prognostic stratification and open new therapeutic alternatives for esophagogastric cancer treatment. Future research on heat shock proteins may improve the treatment outcomes of esophagogastric cancer and allow customized treatment. 

## Figures and Tables

**Table 1:** Main prognostic findings for the heat shock proteins (HSP) overexpression in esophagogastric cancer. (=OS): no change in overall survival; (↓OS): implicates a poorer overall survival; (HOP): HSP 70/HSP 90-organizing protein.

HSP	Esophageal SCC	Esophageal Adenocarcinoma	Gastric Adenocarcinoma
**HSP16.2**	=OS	.	.
**HSP27**	↓ OS	↓ OS	↓ OS
**HSP60**	=OS	.	.
**HSP70**	=OS	↓ OS	↓ OS
**HSP90**	=OS	=OS	=OS
**HOP**	.	.	↓ OS

**Table 2:** The main potential therapies that have already been studied for esophagogastric cancer targeting each class of HSP and their molecular mechanism of action.

HSP Family	HSP Function	HSP Inhibitors
**HSP 27**	Inhibits p53 and p21, and suppresses cellular senescence;	HSP27 inhibitor J2
	Interacts with Akt and blocks the Cyt C and block apoptosis;	
	Associated with EBV infection in gastric cancer;	
	Regulates chemotherapy and radiation response;	
**HSP 40**	Interacts with HSP 70 proteins;	Col003, KNK437
	Regulates p53-mediated apoptosis;	
**HSP 70**	Protects tumor cells from TNF-induced cytotoxicity;	VER-155008, Apoptozole, MKT-077, Pifithrin-μ, CCT251236, HSP70-IN-1, KNK437, YK5, MAL3-101, GRP78-IN-1
	Promotes gastrointestinal tumor proliferation by cell cycle regulation and signaling;	
	Protects gastric cancer cells from apoptosis;	
	HSPA9 (Mortalin) binds to p53 and prevents it from regulating cell cycle apoptosis;	
**HSP 90**	Plays a central role in regulatory pathways such as cell signaling, apoptosis, and cell cycle;	Tanespimycinm, Geldanamycin, Ganetespib, Luminespib, Gamitrinib TPP hexafluorophosphate, Alvespimycin hydrochloride, Pimitespib, Grp94 Inhibitor-1, Onalespib, BIIB021, NVP-HSP990, XL888, Debio 0932, Radicicol, VER-82576, KW-2478, Retaspimycin Hydrochloride, Ethoxyquin, 3-Phenyltoxoflavin, VER-50589, VER-49009, Geldanamycin-FITC, Cucurbitacin D, HS-27, NMS-E973, Gedunin, NCT-58, Alvespimycin, Gamitrinib TPP, YZ129, Cemdomespib, Macbecin, Aminohexylgeldanamycin hydrochloride, HDAC/HSP90-IN-3, 17-AEP-GA, HDAC6/HSP90-IN-1, HSP90-IN-14, MPC-0767, CH5138303, Retaspimycin, Dihydroberberine, HSP90-IN-13, CCT018159, 17-GMB-APA-GA, Tamoxifen-d5, PROTAC HSP90 degrader BP3, Aminohexylgeldanamycin, Chetomin, YK5, Hsp90-IN-15, HSP90-IN-9
	HPV infection seems to be related to HSP90 overexpression in squamous cell carcinoma;	
	Activity of Her2 has been shown to be modulated by molecular chaperones as HSP 90;	
	Contributes to the maturation and stabilization of the telomerase and a large range of oncogenic proteins;	
**HSP 105**	Suppresses stress-induced apoptosis in cancer cells;	KNK437
**HSF1**	Transcription factor that binds to heat shock elements;	NXP800, Rocaglamide, KRIBB11, HM03
	Regulates cell proliferation and turnover;	
	Suppresses apoptosis.	

## Data Availability

No new data were created or analyzed in this study. Data sharing is not applicable to this article.
